# Association of heat shock proteins with all-cause mortality

**DOI:** 10.1007/s11357-012-9417-7

**Published:** 2012-05-04

**Authors:** L. Broer, E. W. Demerath, M. E. Garcia, G. Homuth, R. C. Kaplan, K. L. Lunetta, T. Tanaka, G. J. Tranah, S. Walter, A. M. Arnold, G. Atzmon, T. B. Harris, W. Hoffmann, D. Karasik, D. P. Kiel, T. Kocher, L. J. Launer, K. K. Lohman, J. I. Rotter, H. Tiemeier, A. G. Uitterlinden, H. Wallaschofski, S. Bandinelli, M. Dörr, L. Ferrucci, N. Franceschini, V. Gudnason, A. Hofman, Y. Liu, J. M. Murabito, A. B. Newman, B. A. Oostra, B. M. Psaty, A. V. Smith, C. M. van Duijn

**Affiliations:** 1 Department of Epidemiology, Erasmus Medical Center, Dr. Molewaterplein 50, PO-Box 2040, 3000 CA Rotterdam, The Netherlands; 2 Netherlands Consortium of Healthy Aging, Rotterdam, The Netherlands; 3 Division of Epidemiology and Community Health, School of Public Health, University of Minnesota, Minneapolis, MN USA; 4 Laboratory of Epidemiology, Demography, and Biometry, National Institute on Aging, National Institutes of Health, Bethesda, MD USA; 5 Interfaculty Institute for Genetics and Functional Genomics, Ernst-Moritz-Arndt-University Greifswald, Greifswald, Germany; 6 Department of Epidemiology and Population Health, Albert Einstein College of Medicine, Bronx, NY USA; 7 Department of Biostatistics, Boston University School of Public Health, 715 Albany Street, Talbot Building, Boston, MA 02118 USA; 8 NHLBI’s Framingham Heart Study, Framingham, USA; 9 Clinical Research Branch, National Institute on Aging, Baltimore, MD USA; 10 California Pacific Medical Center, San Francisco, CA USA; 11 Department of Society, Human Development, and Health, Harvard School of Public Health, 677 Huntington Avenue, Boston, MA 02115 USA; 12 Department of Biostatistics, University of Washington, Seattle, WA USA; 13 Institute for Aging Research and the Diabetes Research Center, Albert Einstein College of Medicine, Bronx, NY USA; 14 Department of Medicine, Albert Einstein College of Medicine, Bronx, NY USA; 15 Department of Genetics, Albert Einstein College of Medicine, Bronx, NY USA; 16 Institute of Community Medicine, Ernst-Moritz-Arndt-University Greifswald, Greifswald, Germany; 17 Hebrew Senior Life Institute for Aging Research and Harvard Medical School, Boston, MA USA; 18 Dental School, Ernst-Moritz-Arndt-University Greifswald, Greifswald, Germany; 19 Sticht Center on Aging, Wake Forest University School of Medicine, Winston-Salem, NC USA; 20 Medical Genetics Institute, Cedars-Sinai Medical Center, Los Angeles, CA USA; 21 Department of Psychiatry, Erasmus Medical Center, Rotterdam, The Netherlands; 22 Department of Internal Medicine, Erasmus Medical Center, Rotterdam, The Netherlands; 23 Institute of Clinical Chemistry and Laboratory Medicine, University Medicine Greifswald, Ernst-Moritz-Arndt-University Greifswald, Greifswald, Germany; 24 Geriatric Unit, Azienda Sanitaria Firenze (ASF), Florence, Italy; 25 Department of Internal Medicine B, University Medicine Greifswald, Greifswald, Germany; 26 Department of Epidemiology, University of North Carolina, Chapel Hill, NC USA; 27 Icelandic Heart Association, Kópavogur, Iceland; 28 University of Iceland, Reykjavik, Iceland; 29 Department of Biostatistical Sciences, Wake Forest University School of Medicine, Winston-Salem, NC USA; 30 Section of General Internal Medicine, Department of Medicine, Boston University School of Medicine, 72 E. Concord Street, Boston, MA 02118 USA; 31 Graduate School of Public Health, University of Pittsburgh, Pittsburgh, PA USA; 32 Department of Clinical Genetics, Erasmus Medical Center, Rotterdam, The Netherlands; 33 Cardiovascular Health Research Unit, Departments of Medicine, Epidemiology, and Health Services, University of Washington, Seattle, WA USA; 34 Group Health Research Unit, Group Health Cooperative, Seattle, WA USA

**Keywords:** Heat shock proteins, HEAT shock factor 2, All-cause mortality

## Abstract

Experimental mild heat shock is widely known as an intervention that results in extended longevity in various models along the evolutionary lineage. Heat shock proteins (HSPs) are highly upregulated immediately after a heat shock. The elevation in HSP levels was shown to inhibit stress-mediated cell death, and recent experiments indicate a highly versatile role for these proteins as inhibitors of programmed cell death. In this study, we examined common genetic variations in 31 genes encoding all members of the HSP70, small HSP, and heat shock factor (HSF) families for their association with all-cause mortality. Our discovery cohort was the Rotterdam study (RS1) containing 5,974 participants aged 55 years and older (3,174 deaths). We assessed 4,430 single nucleotide polymorphisms (SNPs) using the HumanHap550K Genotyping BeadChip from Illumina. After adjusting for multiple testing by permutation analysis, three SNPs showed evidence for association with all-cause mortality in RS1. These findings were followed in eight independent population-based cohorts, leading to a total of 25,007 participants (8,444 deaths). In the replication phase, only *HSF2* (rs1416733) remained significantly associated with all-cause mortality. Rs1416733 is a known cis-eQTL for *HSF2*. Our findings suggest a role of *HSF2* in all-cause mortality.

**Electronic supplementary material:**

The online version of this article (doi:10.1007/s11357-012-9417-7) contains supplementary material, which is available to authorized users.

## Introduction

Experimental mild heat shock is widely known as an intervention that results in extended longevity (Cypser and Johnson 2001). Brief exposure to elevated heat resulted in a 15 % increase in the mean life span of *Caenorhabditis elegans*, compared to non heat-shocked controls (Cypser and Johnson 2001; Cypser and Johnson 2002; Lithgow et al. 1995). Similar effects have also been seen in *Drosophila melanogaster* (Hercus et al. 2003; Le Bourg et al. 2001), in yeast (Shama et al. 1998), and in cultured human cells (Rattan 1998). In the early 1960s, a group of proteins, now known as heat shock proteins (HSPs) were discovered, which were highly upregulated immediately after a heat shock (Ritossa 1962, 1996). Whether HSPs are responsible for longevity is still under debate, as their levels are only elevated for a short period of time after a heat shock (Link et al. 1999). However, the elevation in HSP levels during the heat shock response was shown to inhibit stress-mediated cell death, and recent experiments indicate a highly versatile role for these proteins as inhibitors of programmed cell death (Garrido et al. 2006).

HSPs can be subdivided in several smaller families, including HSP90, HSP70, HSP60, HSP40, small HSP (sHSP), and HSP10 (Kampinga et al. 2009). From these families, HSP70 and sHSPs show an association with longevity. In *C. elegans*, extra copies of a homolog of *HSPA9* (member of HSP70), otherwise known as mortalin, extended life span up to 45 % (Yokoyama et al. 2002). In humans, decreased serum levels of HSP70 have been associated with exceptional longevity (95+) (Terry et al. 2006). However, the same study evaluated two single nucleotide polymorphisms (SNPs) in *HSPA1A* and *HSPA1B* which were not found to be associated to exceptional longevity (Terry et al. 2006).

The over-expression of members of the sHSP family has been shown to extend life of *C. elegans* and *D. melanogaster* by up to 32 % (Morrow et al. 2004b; Walker et al. 2001). Conversely, the absence of expression of a sHSP member decreases lifespan of *D. melanogaster* by 40 % (Morrow et al. 2004a).

HSP expression is regulated by a group of transcription factors known as heat shock factors (HSFs), of which HSF1 is considered to be the master-switch of HSP expression (Akerfelt et al. 2010). Strong evidence exists for a highly important role for HSF1 in longevity. Reduced activity of HSF1 in *C. elegans* leads to a rapid aging phenotype with a markedly reduced lifespan of 60 % (Garigan et al. 2002). Conversely, animals with an additional HSF1 gene copy lived approximately 40 % longer than normal (Hsu et al. 2003). A strong relationship was found between HSF1 and DAF-16, which functions in the *C. elegans* insulin/IGF-1 signaling pathway (Hsu et al. 2003). Both genes were shown to function, at least in part, by increasing sHSP gene expression (Hsu et al. 2003).

We have tested 31 genes encoding all members of the HSP70, sHSP, and HSF families and assessed their association with all-cause mortality. To our knowledge, this is the first large-scale candidate gene study of these HSPs and their association to all-cause mortality to be performed.

## Methods

### Discovery study

Our discovery cohort was the Rotterdam study (RS1). RS1 is a population-based cohort study that investigates the occurrence and determinants of diseases in the elderly (Hofman et al. 2011). Baseline examinations, including a detailed questionnaire, physical examination, and blood collection, were conducted between 1990 and 1993. The Medical Ethics Committee at Erasmus Medical Center approved the study protocol. All of the participants were followed for incident diseases through linkage to the general practitioner data base and record review by trained medical investigators. General practitioners' hospital records as well as death certificates were used for identification of deaths (all-cause mortality) through January 1, 2009.

Genomic DNA was extracted from whole blood samples using standard methods (Miller et al. 1988). Genome-wide SNP genotyping was performed using Infinium II assay on the HumanHap550 Genotyping BeadChips (Illumina Inc, San Diego, USA). Approximately two million SNPs were imputed using release 22 HapMap CEU population as reference. The imputations were performed using MACH software (http://www.sph.umich.edu/csg/abecasis/MACH/). The quality of imputations were checked by contrasting imputed and actual genotypes at 78,844 SNPs not present on Illumina 550K for 437 individuals for whom these SNPs were directly typed using Affymetrix 500K. Using the “best guess” genotype for imputed SNPs the concordance rate was 99 % for SNPs with *R* ^2^ (ratio of the variance of imputed genotypes to the binomial variance) quality measure greater than 0.9; concordance was still over 90 % (94 %) when *R* ^2^ was between 0.5 and 0.9. The GWAS of all-cause mortality has been analyzed and is published elsewhere (Walter et al. 2011).

For the study of HSPs presented here, a total of 4,430 SNPs in 31 genes were initially selected for the association test on the basis of the following criteria: (1) position within the genes of interest with a margin of 100 kb on each side of the genes according to NCBI build 36.3; (2) *p* value for Hardy–Weinberg equilibrium test ≥0.0001; and (3) call rate ≥95 %. For further analysis and selection of SNPs for replication analysis, only SNPs with an *R* ^2^ higher than 0.8 and a minor allele frequency higher than 0.05 were selected.

### Analyses

We performed single SNP analyses using ProbABEL(Aulchenko et al. 2010). We used survival analysis (semi-parametric Cox proportional hazard model), adjusted for age at DNA blood collection and for sex, to model continuous time to death in individuals that were older than 55 years at baseline. To calculate empirical significance for SNPs, permutations were performed per region of interest (ROI) (Churchill and Doerge 1994; Fisher 1935). Briefly, the empirical distribution of the region-wide maximum of the test statistic under the null was obtained in 10,000 replications. To estimate empirical significance, each observed test statistic was compared with null statistics obtained empirically and the *p* value was estimated as the proportion of replicas generating the test statistics greater than or equal to the observed statistic. The permutation analysis keeps the original genotypes for each individual, but randomly allocates the phenotypes for each consecutive permutation. Therefore, the linkage disequilibrium structure of genes is not broken up. We did not perform any additional correction for the number of ROIs after permutations as we used the permutation analysis to significantly reduce the number of SNPs to be selected for validation of the effect of the SNP. For each suggestively associated ROI (*p*_permuted_ < 0.10), we next selected the “truly associated” SNPs in a backward stepwise survival analysis until only nominally significant SNPs remained in the model. These independently associated SNPs were then followed up in eight independent cohorts. As effect estimates are expected to be small, a strict replication based on the *p* value and hazard ratio (HR) observed in individual replication studies, might be difficult to achieve due to power issues. Therefore, we evaluated whether a SNP was showing a HR in the same direction in all cohorts, i.e., whether findings were consistent over the various cohorts. Similar to genome-wide association studies, we performed a joint meta-analysis of the discovery and the replication samples and tested whether the joint *p* value was significant using a Bonferroni correction for the number of SNPs validated in the replication phase.

We used four studies that are part of the Cohorts for Heart and Aging Research in Genomic Epidemiology (CHARGE) consortium (Psaty et al. 2009) plus an additional four associated cohorts to evaluate whether findings were consistent. The studies included in this replication are: Cardiovascular Health Study (CHS); Framingham Heart Study (FHS); Atherosclerosis Risk Communities Study (ARIC); Age, Gene/Environment Susceptibility–Reykjavik Study (AGES); Health, Aging and Body Composition (HABC); Baltimore Longitudinal Study of Ageing (BLSA); InCHIANTI (ICH); and Study of Health in Pomerania (SHIP). All studies are longitudinal population-based studies periodically assessing the health and vital status of their participants. All participants included in this analysis were at least 55 years of age at the time of blood draw for DNA and provided written informed consent. Detailed information on the replication studies can be found in the supplement.

## Results

General characteristics of the discovery cohort as well as the eight replication cohorts are shown in Table 1. In the discovery cohort, there were 3,174 deaths with a mean age at death of 83.2 and mean follow-up of 12.5 years. In RS1 and replications combined, there were 8,444 deaths with a mean age at death of 81.1. The mean follow-up time ranged from 5.2 to 15.7 years. A summary of all SNPs tested in the HSP genes in RS1 can be found in Supplementary Table 1. Three HSP70 genes are located head to head on chromosome 6 and are taken as one ROI with 148 SNPs (HSPA1). Similarly, two sHSP genes on chromosome 11 with 66 SNPs were located nearby each other and taken as one ROI (ROIchr11). Also of note is that the HSPs are currently undergoing a change in nomenclature (Kampinga et al. 2009). Supplementary Table 1 provides both the official gene names and the gene names according to the new nomenclature. Here, we will use the aliases from the new nomenclature.

**Table 1:** General characteristics

Study	*N*	*N* deceased	Mean age at Baseline (±SD)	Mean age at death (±SD)	Sex, % female	Mean follow-up time in years (±SD)
Rotterdam study (RS1)	5974	3,174	69.4 (9.1)	83.2 (8.3)	59	12.5 (5.2)
Cardiovascular health study (CHS)	3,267	1,718	72.3 (5.4)	83.4 (6.3)	61	12.3 (4.2)
Framingham heart study (FHS)	3,136	654	70.0 (10.2)	83.0 (9.3)	56	6.0 (2.4)
Atherosclerosis risk communities study (ARIC)	4,511	1,108	59.4 (2.9)	71.3 (5.4)	50	15.7 (3.7)
Age, gene/environment susceptibility-Reykjavik study (AGES)	3,219	558	76.4 (5.5)	79.3 (5.9)	58	5.2 (1.3)
The health, aging and body composition (HABC)	1,661	460	73.8 (2.8)	80.4 (3.7)	47	8.2 (2.3)
Baltimore longitudinal study of ageing (BLSA)	620	183	62.0 (8.8)	86.8 (8.0)	41	15.7 (8.2)
Invecchiare nel Chianti (InCHIANTI)	902	183	72.5 (7.7)	85.4 (7.9)	56	5.9 (0.9)
Study of health in Pomerania (SHIP)	1,717	406	66.4 (7.2)	76.9 (7.2)	47	9.2 (2.4)
TOTAL	25,007	8,444	69.0 (8.9)	81.1 (8.4)	55	10.6 (5.4)

**Fig. 1:**
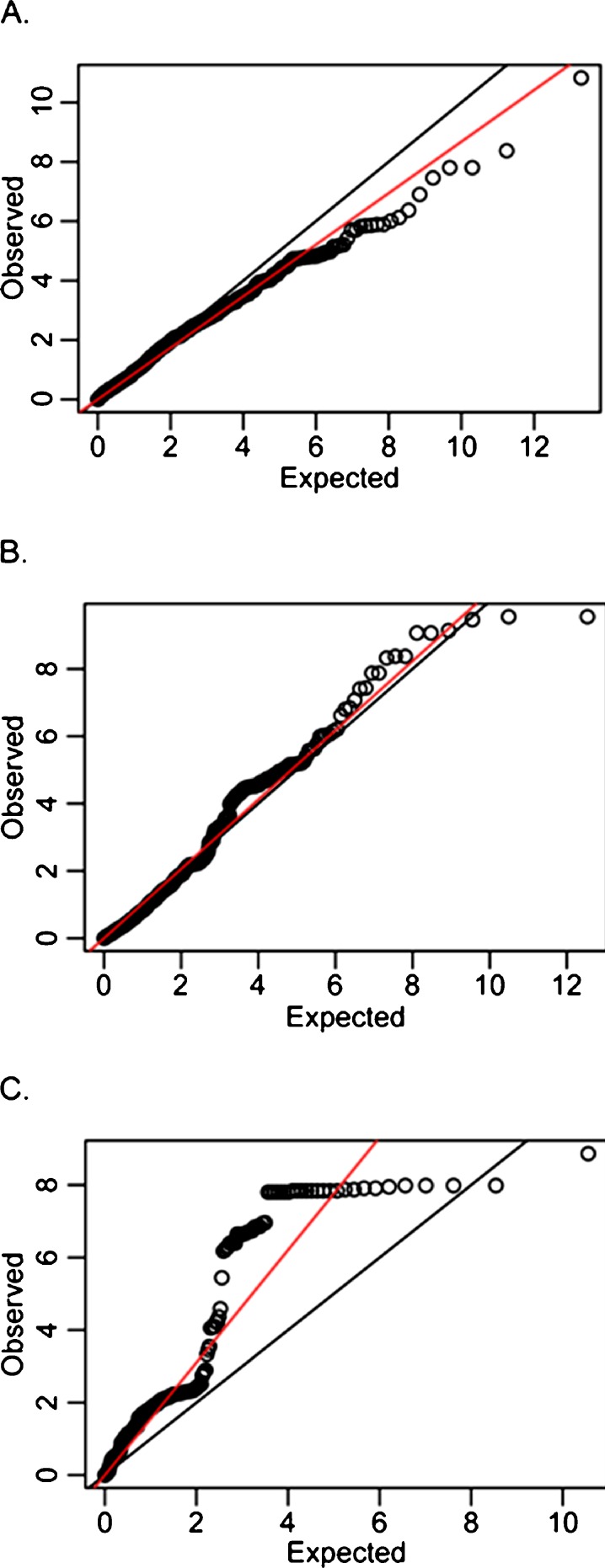
Observed versus expected *p* value plot for the SNPs tested in the Rotterdam study (chi^2^ are given at the x-axis and y-axis). **a** HSP70; **b** sHSP; **c** HSF. *Black line* depicts the expected findings; *red line* depicts the observed ones

**Fig. 2:**
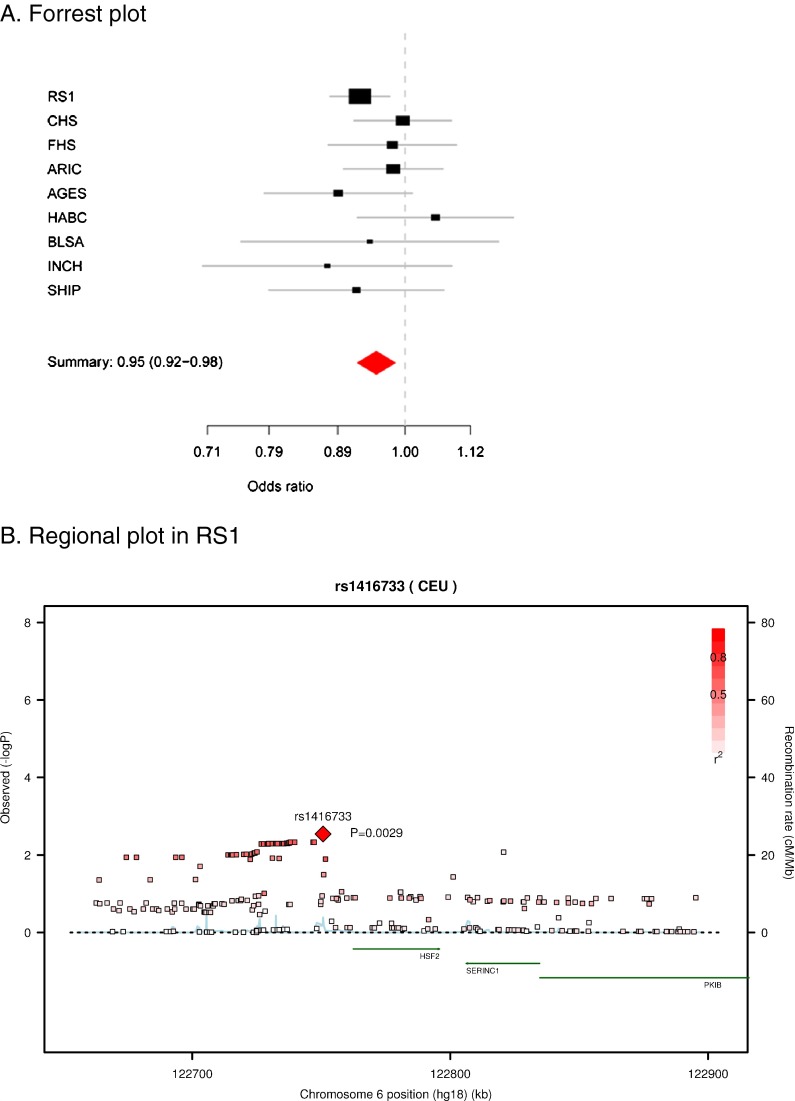
The meta-analysis of the HSF2 rs1416733 SNP in the nine population cohorts. **a** Forrest plot. **b** Regional plot in RS1

Figure 1 a–d illustrate the observed versus the expected chi^2^ of the SNPs tested in the different HSP gene families in RS1. The plots show an excess of low *p* values for tested SNPs in sHSP (1b) and HSF (1c) but not for HSP70 (1a). Supplementary Fig. 1 shows an overview of the *p* values in all HSP70, sHSP, and HSF genes tested in RS1. After adjusting for multiple testing by permutation analysis, three genes showed evidence for association to mortality in RS1. These genes encode HSP70 member *HSPA8*, sHSP member *HSPB1*, and HSF member *HSF2*. Table 2 shows the three SNPs that were selected for replication. In the replication phase, only one SNP reached a Bonferroni adjusted *p* value for significance (0.05/3 = 0.017). Figure 2a shows the Forest plot of the meta-analysis of *HSF2* rs1416733. The direction of effect was consistent across populations with only one study (HABC) showing an opposite HR. The summary HR for this SNP was 0.95 (95 % CI 0.92–0.98; *p* = 0.003) for the A allele. When excluding the discovery cohort from the meta-analysis, the HR remained virtually unchanged (0.97: CI 0.93–1.00). In Fig. 2b, a regional plot of all SNPs tested in HSF2 from the original RS1 cohort can be found. In the plot, we can see that rs1416733 is located 11.7 Kb from the 5′ region of the *HSF2* gene, with no other genes nearby.

**Table 2:** Results of single SNP replication

					RS1				CHARGE meta-analysis				
Family	Gene	SNP	Non-coded Allele	Coded Allele	Frequency coded allele	HR	95 % CI	*p* value	Frequency coded allele	HR	95 % CI	Study Effect Direction	*p* value
HSP70	HSPA8	rs12574703	G	A	0.05	1.21	1.08–1.36	0.001	0.05	1.07	0.99–1.14	+ − + − ++++ −	0.080
sHSP	HSPB1	rs7797781	C	T	0.79	0.91	0.86–0.97	0.002	0.78	0.96	0.93–1.00	−− + − +++++	0.035
HSF	**HSF2**	rs1416733	G	A	0.35	0.92	0.88–0.97	0.003	0.35	0.95	0.92–0.98	−−−−− + −−−	0.003

*HR* Hazard ratios and *95 % CI* 95 % confidence interval are for each additional coded allele. Study effect direction: study-specific information is presented in the order: RS1, CHS, FHS, ARIC, AGES, HABC, BLSA, ICH, and SHIP. Direction: “+” stands for “HR greater than 1”; “−” stands for “HR smaller than 1”. *Bold* shows which genes passed Bonferroni adjusted *p* value for significance (0.05/3 = 0.017)

## Discussion

In our study, we found a significant association between *HSF2* and all-cause mortality. The top associated variant, rs1416733, is located 11.7 Kb from the 5′ region of the *HSF2* gene on chromosome 6 and is a known cis-eQTL for *HSF2* (Zeller et al. 2010). Each additional copy of the A allele for SNP rs1416733 increased lifespan with a HR of 0.95 in a meta-analysis of nine independent cohort studies. This effect was consistent in direction in eight out of nine cohorts.

The HSF family, like most HSP families, is a highly conserved family across species, indicating a vital role for the survival of the respective organism (Liu et al. 1997). Unlike many other HSP families, there is virtually no redundancy in the HSF family, with HSF1 as the most crucial family member (Akerfelt et al. 2010). The importance of HSF1 is advocated strongly by the large effects of genetic mutations in this gene on longevity in *C. elegans* (Garigan et al. 2002; Hsu et al. 2003). These large effects are not anticipated in human research, which could explain why we find no evidence for association of SNPs in *HSF1* with all-cause mortality (Walter et al. 2011). HSF2 is less known in longevity research. It has previously been mainly described for its role as a development- and differentiation-specific factor (Kallio et al. 2002; Wang et al. 2003). The role of HSF2 in later life has remained unknown for a long time (Wu 1995). Recently, evidence is emerging to suggest that HSF2 modulates HSF1 activity (Akerfelt et al. 2010). It has been shown that HSF2 activation leads to activation of HSF1, revealing a functional interdependency (Sandqvist et al. 2009). It has been proposed that heterotrimerization of HSF1 and HSF2 integrates transcriptional activation in response to distinct stress and developmental stimuli (Sandqvist et al. 2009). Additionally, though HSF2 was never considered to be heat-inducible, a recent study shows that a mild heat-shock in the physiological range does activate HSF2 and has a significant impact on the proteostasis of the cell (Shinkawa et al. 2011).

We found no evidence for association of HSP70 with all-cause mortality. HSP70 is the only HSP family for which SNPs in a couple of genes have been studied for longevity in humans previously. However, these studies only studied two or three SNPs and only in the heat-inducible members of HSP70, namely *HSPA1A*, *HSPA1B*, and *HSPA1L*, all located on chromosome 6 (Altomare et al. 2003; Ross et al. 2003; Terry et al. 2006). In our study, no association was found for these three genes with longevity. We add a more thorough investigation of these genes and the other HSP70 genes by extensively covering common variance.

The sHSPs have been named most often in animal studies for their relationship with longevity (Morrow et al. 2004a; Morrow et al. 2004b; Walker et al. 2001). However, we find no significant associations for single SNPs in these genes. Off course, what we find in animal studies does not always translate well to humans. For example, though genetic variations in IGF-1 signaling have been found in humans, they do not have nearly as strong effects on longevity as in animal models (Kuningas et al. 2008). Another study has found that mutations in HSF1 lead to upregulated sHSP expression in *C. elegans* (Hsu et al. 2003). We cannot exclude that genetic variation in *HSF2* activates a similar mechanism in humans through its effect on HSF1.

Our study has a major advantage since the discovery cohort—the Rotterdam Study—is a large, population-based study. Further, the eight replication cohorts are also relatively large, established population-based epidemiological cohorts. A limitation in the interpretation of our data is that our findings were significant in the meta-analysis but did not reach significance in the individual cohorts except for the RS1 cohort; however, a total of eight out of nine studies showed an effect in the same direction for *HSF2*. This could be explained by the markedly smaller percentage of deaths in most replication cohorts (except CHS) compared to the discovery cohort. The number of deceased is critical for the statistical power of the study. Even though some of these studies had a longer mean follow-up time (ARIC and BLSA), the population in these studies were on average younger at baseline compared to RS1, which explains the fewer number of deaths and leaves the individual replication studies underpowered to identify rs1416733.

Until now, no large-scale studies have been performed investigating the role of HSP70, sHSP, or HSF genes in all-cause mortality in humans. In our candidate gene study in nine population-based cohorts, we found significant evidence suggesting that genetic variants in *HSF2* are associated with all-cause mortality. Combining these data with those of earlier functional studies, in particular in *C. elegans*, makes it likely that *HSF2* plays a role in human all-cause mortality.

## Electronic supplementary material

Below is the link to the electronic supplementary material.

ESM 1 (DOC 398 kb)
