# Supplementary material for: Association of heat shock proteins with all-cause mortality
Source: Age (Dordr). 2012 May 4;35(4):1367–76. doi: 10.1007/s11357-012-9417-7 (PMC3705092; doi:10.1007/s11357-012-9417-7)
Supplement: Supplementary file 1 — (DOC 398 kb) [file 11357_2012_9417_MOESM1_ESM.doc]

# Association of Heat Shock Proteins with mortality

L. Broer^1,2^, E.W. Demerath^3^, M.E. Garcia^4^, G. Homuth^5^, R.C. Kaplan^6^, K.L. Lunetta^7,8^, T. Tanaka^9^, G.J. Tranah^10^, S. Walter^11^, A.M. Arnold^12^, G. Atzmon^13,14,15^, T.B. Harris^4^, W. Hoffmann^16^, D. Karasik^8,17^, D.P. Kiel^8,17^, T. Kocher^18^, L.J. Launer^4^, K.K. Lohman^19^, J.I. Rotter^20^, H. Tiemeier^1,2,21^, A.G. Uitterlinden^1,22^, H. Wallaschofski^23^, S. Bandinelli^24^, M. Dörr^25^, L. Ferrucci^9^, N. Franceschini^26^, V. Gudnason^27,28^, A. Hofman^1^, Y. Liu^29^, J.M. Murabito^8,30^, A.B. Newman^31^, B.A. Oostra^32^, B.M. Psaty^33,34^, A.V. Smith^27,28^, C.M. van Duijn^1,2^

**Affiliations**

1. Department of Epidemiology, Erasmus Medical Center, Rotterdam, The Netherlands
2. Netherlands Consortium of Healthy Aging, Rotterdam, The Netherlands
3. Division of Epidemiology and Community Health, School of Public Health, University of Minnesota, Minneapolis, Minnesota, USA
4. Laboratory of Epidemiology, Demography, and Biometry, National Institute on Aging, National Institutes of Health, Bethesda, MD, USA
5. Interfaculty Institute for Genetics and Functional Genomics, Ernst-Moritz-Arndt-University Greifswald, Greifswald, Germany
6. Department of Epidemiology and Population Health, Albert Einstein College of Medicine, Bronx NY, USA
7. Department of Biostatistics,Boston University School of Public Health
8. NHLBI's Framingham Heart Study
9. Clinical Research Branch, National Institute on Aging, Baltimore, MD, USA
10. California Pacific Medical Center, San Francisco, CA, USA
11. Department of Society, Human Development, and Health, Harvard School of Public Health
12. Department of Biostatistics, Unversity of Washington, Seattle, WA, USA
13. Institute for Aging Research and the Diabetes Research Center. Albert Einstein College of Medicine, Bronx, NY, USA
14. Department of Medicine Albert Einstein College of Medicine, Bronx, NY, USA
15. Department of Genetic Albert Einstein College of Medicine, Bronx, NY, USA
16. Institute of Community Medicine, Ernst-Moritz-Arndt-University Greifswald, Greifswald, Germany
17. Hebrew SeniorLife Institute for Aging Research and Harvard Medical School, Boston, Massachusetts, USA
18. Dental School, Ernst-Moritz-Arndt-University Greifswald, Greifswald, Germany
19. Sticht Center on Aging, Wake Forest University School of Medicine, Winston-Salem, NC, USA
20. Medical Genetics Institute, Cedars-Sinai Medical Center, Los Angeles, CA, USA
21. Department of Psychiatry, Erasmus Medical Center, Rotterdam, The Netherlands
22. Department of Internal Medicine, Erasmus Medical Center, Rotterdam, The Netherlands
23. Institute of Clinical Chemistry and Laboratory Medicine, University Medicine Greifswald,Ernst-Moritz-Arndt-University Greifswald,Greifswald, Germany
24. Geriatric Unit, Azienda Sanitaria Firenze (ASF), Florence, Italy
25. Greifswald Zentrum für innere medizin, Klinik und Poliklink für Innere Medizin B, Ernst-Moritz-Arndt-University Greifswald, Greifswald, Germany
26. Department of Epidemiology, University of North Carolina, Chapel Hill, NC, USA
27. Icelandic Heart Association, Kópavogur, Iceland
28. University of Iceland, Reykjavik, Iceland
29. Department of Biostatistical Sciences, Wake Forest University School of Medicine, Winston-Salem, NC, USA
30. Section of General Internal Medicine, Department of Medicine, Boston University School of Medicine
31. Graduate School of Public Health, University of Pittsburgh, Pittsburgh, PA, USA
32. Department of Clinical Genetics, Erasmus Medical Center, Rotterdam, The Netherlands
33. Cardiovascular Health Research Unit, Departments of Medicine, Epidemiology, and Health Services, University of Washington, Seattle WA, USA
34. Group Health Research Unit, Group Health Cooperative, Seattle, WA, USA

Corresponding author:

CM van Duijn, Department of Epidemiology, Erasmus University Medical Center, Dr. Molewaterplein 50, PO-Box 2040, 3000 CA Rotterdam, The Netherlands

Tel: (+31) 10-7043394, Fax: (+31) 10-7044657, Email: [c.vanduijn@erasmusmc.nl](mailto:c.vanduijn@erasmusmc.nl)

**Supplementary methods:**

The Cardiovascular Health Study (CHS) is a prospective population-based cohort study of CVD, mortality, and other outcomes in 5888 65+ year old Medicare-eligible adults living in four US communities (1). Recruitment of the initial predominantly white cohort was completed in 1990 and 3,267 participants fulfilled the inclusion criteria of this study and had genotyping information available. Only European or European Americans were included in the analysis, so the CHS African Americans were excluded. Major incident health events and deaths were identified through several methods, including: questionnaires completed by participants at each semi-annual contact during follow-up; reports by family members; and periodic searches of the Medicare Utilization database, the National Death Index, and local newspaper obituaries through 30.06.2005.

The Framingham Heart Study (FHS) was initiated to study determinants of CVD and other major illnesses. The original cohort was recruited in 1948 and the offspring of the original cohort participants and offspring spouses were enrolled in 1971 (2-3). DNA was obtained for genetic studies in the 1990s from surviving Original cohort and Offspring participants. The exam at which DNA was obtained is considered the baseline exam for these analyses. For 3136 participants genetic information was available and the eligibility criteria for this analysis were met. All participants remain under continuous surveillance and deaths and incident diseases that occurred through 01.01.2007 were included.

Atherosclerosis Risk Communities Study (ARIC), initiated in 1987-89, is a population-based cohort study of cardiovascular disease and its risk factors sponsored by the National Heart, Lung and Blood Institute (NHLBI) (4). 4511 participants aged 55 or older fulfilled the inclusion criteria of this study and had genotyping information available. Follow-up for clinical events was obtained through annual surveillance to ascertain vital status as well as cardiovascular events, including hospitalizations and deaths, adjudicated by an Events Committee, and complete until 31.12.2005.

The Age, Gene/Environment Susceptibility -Reykjavik Study (AGES) was initiated to examine potential genetic susceptibility and gene/environment interaction (5). Between 2002 and 2006, baseline exams were conducted in survivors from the Reykjavik Study. 3219 AGES-Reykjavik participants were eligible for participation and with available genotype information. Follow-up information was complete till 27.04.2009 via linkage to electronic medical records and vital status registry.

The Health, Aging and Body Composition (HABC) Study is a prospective cohort study of community-dwelling black and white men and women living in Memphis, TN, or Pittsburgh, PA, aged 70-79 years at recruitment in 1997. 1661 white participants were eligible to participate in this analysis and had genotyping information available. Surveillance was conducted by in person examination alternating with a telephone interview every 6 months. Hospital records, death certificates, and autopsy data were reviewed by committee to adjudicate causes of death and complete through 26.11.2007.

The Baltimore Longitudinal Study of Ageing (BLSA) study is a population-based study aimed to evaluate contributors of healthy aging in the older population residing predominantly in the Baltimore-Washington DC area. The study began in 1958, and follow up data through to 20.12.2004 for 599 subjects was used for this analysis. Participants returned for follow-up visits every 1-2 years where presence of chronic disease was assessed by a physician during the physical exam, self-report or medication use. Death was ascertained through death certificates or report by family members.

The InCHIANTI (ICH) study is a population-based epidemiological study aimed at evaluating the factors that influence mobility in the older population living in the Chianti region in Tuscany, Italy that was initiated in 1998. Presence of chronic diseases was ascertained by a combination of assessments by trained geriatrician, medication use, blood tests, and self-reported physician diagnosis. Death was determined using a death registry. 686 subjects with genotype and mortality and disease data assessed through to 06.03.2006 were used for this analysis.

The Study of Health in Pomerania (SHIP) is a cross-sectional survey in West Pomerania, the north-east area of Germany initiated in 1997 (6-7). 1717 participants were eligible to participate in this study and had genotyping information available. Information on vital status was acquired at annual intervals from the local health authority and complete through 03.11.2009.

**References supplementary methods:**

1. Fried LP, Borhani NO, Enright P, Furberg CD, Gardin JM, Kronmal RA, et al. The Cardiovascular Health Study: design and rationale. Ann Epidemiol. 1991 Feb;1(3):263-76.

2. Dawber TR, Kannel WB. The Framingham study. An epidemiological approach to coronary heart disease. Circulation. 1966 Oct;34(4):553-5.

3. Kannel WB, Feinleib M, McNamara PM, Garrison RJ, Castelli WP. An investigation of coronary heart disease in families. The Framingham offspring study. Am J Epidemiol. 1979 Sep;110(3):281-90.

4. The Atherosclerosis Risk in Communities (ARIC) Study: design and objectives. The ARIC investigators. Am J Epidemiol. 1989 Apr;129(4):687-702.

5. Harris TB, Launer LJ, Eiriksdottir G, Kjartansson O, Jonsson PV, Sigurdsson G, et al. Age, Gene/Environment Susceptibility-Reykjavik Study: multidisciplinary applied phenomics. Am J Epidemiol. 2007 May 1;165(9):1076-87.

6. John U, Greiner B, Hensel E, Ludemann J, Piek M, Sauer S, et al. Study of Health In Pomerania (SHIP): a health examination survey in an east German region: objectives and design. Soz Praventivmed. 2001;46(3):186-94.

7. Volzke H, Alte D, Schmidt CO, Radke D, Lorbeer R, Friedrich N, et al. Cohort Profile: The Study of Health in Pomerania. Int J Epidemiol. 2010 Feb 18.

**Supplementary Figures**

Supplementary Figure 1: -log(p-values) of all SNPs tested. Each gene tested is a bar. Order: HSP70, sHSP, HSF





Horizontal lines: p-values of 0.05, 0.01 and 0.001 respectively.

Vertical lines: border between Heat Shock Protein (HSP) families. First part are the HSP70 genes, second part are the small HSP genes, last part are the Heat Shock Factor genes.

**Supplementary Tables**

Supplementary Table 1: overview of all genes tested in RS1 and the number of SNPs per gene

| *Official gene name* | *HSP nomenclature* | *Family* | *Chr* | *Start gene* | *End gene* | *ROI* | *Total SNPs* |
| --- | --- | --- | --- | --- | --- | --- | --- |
| HSPA6 | HSPA6 | HSP70 | 1 | 159760660 | 159763311 | HSPA6 | 139 |
| HSPA4L | **HSPH3** | HSP70 | 4 | 128922903 | 128973976 | HSPH3 | 84 |
| HSPA4 | **HSPH2** | HSP70 | 5 | 132415561 | 132468608 | HSPH2 | 138 |
| HSPA9 | HSPA9 | HSP70 | 5 | 137918923 | 137939014 | HSPA9 | 129 |
| HSPA1L | HSPA1L | HSP70 | 6 | 31885375 | 31890786 | HSPA1 | 148 |
| HSPA1A | HSPA1A | HSP70 | 6 | 31891316 | 31893698 |
| HSPA1B | HSPA1B | HSP70 | 6 | 31903503 | 31906010 |
| HSPA5 | HSPA5 | HSP70 | 9 | 127036953 | 127043430 | HSPA5 | 79 |
| HSPA14 | HSPA14 | HSP70 | 10 | 14920267 | 14953746 | HSPA14 | 161 |
| HSPA12A | HSPA12A | HSP70 | 10 | 118419930 | 118492786 | HSPA12A | 304 |
| HYOU1 | **HSPH4** | HSP70 | 11 | 118420110 | 118433122 | HSPH4 | 122 |
| HSPA8 | HSPA8 | HSP70 | 11 | 122433410 | 122438054 | HSPA8 | 218 |
| HSPH1 | HSPH1 | HSP70 | 13 | 30608762 | 30634117 | HSPH1 | 263 |
| HSPA2 | HSPA2 | HSP70 | 14 | 64072376 | 64079708 | HSPA2 | 118 |
| HSPA12B | HSPA12B | HSP70 | 20 | 3661356 | 3681758 | HSPA12B | 203 |
| HSPA13 | HSPA13 | HSP70 | 21 | 14665307 | 14677380 | HSPA13 | 277 |
| HSPB7 | HSPB7 | sHSP | 1 | 16213111 | 16217538 | HSPB7 | 141 |
| HSPB11 | HSPB11 | sHSP | 1 | 54159822 | 54183876 | HSPB11 | 131 |
| HSPB3 | HSPB3 | sHSP | 5 | 53787202 | 53787964 | HSPB3 | 252 |
| HSPB1 | HSPB1 | sHSP | 7 | 75769859 | 75771548 | HSPB1 | 100 |
| ODF1 | **HSPB10** | sHSP | 8 | 103633036 | 103642422 | HSPB10 | 252 |
| CRYAB | **HSPB5** | sHSP | 11 | 111284560 | 111287683 | ROIchr11 | 66 |
| HSPB2 | HSPB2 | sHSP | 11 | 111288709 | 111290027 |
| HSPB8 | HSPB8 | sHSP | 12 | 118100978 | 118116934 | HSPB8 | 296 |
| HSPB9 | HSPB9 | sHSP | 17 | 37528395 | 37528874 | HSPB9 | 59 |
| HSPB6 | HSPB6 | sHSP | 19 | 40937310 | 40939770 | HSPB6 | 95 |
| CRYAA | **HSPB4** | sHSP | 21 | 43462210 | 43465982 | HSPB4 | 158 |
| HSF2 | HSF2 | HSF | 6 | 122762494 | 122795810 | HSF2 | 253 |
| HSF1 | HSF1 | HSF | 8 | 145486088 | 145509188 | HSF1 | 25 |
| HSF4 | HSF4 | HSF | 16 | 65754858 | 65761349 | HSF4 | 79 |
| HSF5 | HSF5 | HSF | 17 | 53852528 | 53920758 | HSF5 | 140 |
| **Total** | (31 genes) |  |  |  |  |  | 4430 |

Genes that are bold in second column are genes undergoing a name change in accordance to the new HSP nomenclature.
